# Comparison of Microplastic Pollution in Beach Sediment and Seawater at UNESCO Can Gio Mangrove Biosphere Reserve

**DOI:** 10.1002/gch2.202100044

**Published:** 2021-07-16

**Authors:** Vo Thi Kim Khuyen, Dinh Vu Le, Axel René Fischer, Christina Dornack

**Affiliations:** ^1^ Institute of Waste Management and Circular Economy Department of Hydrosciences Faculty of Environmental Sciences Technische Universität Dresden Pratzschwitzer Str. 15 01796 Pirna Germany; ^2^ Faculty of Chemical Engineering Industrial University of Ho Chi Minh City 12 Nguyen Van Bao Str., Go Vap Dist. Ho Chi Minh City 70000 Vietnam

**Keywords:** Can Gio beach, Cape of Dong Tranh, microplastics, Raman spectroscopy, sand sediment, seawater, tourism

## Abstract

Microplastics have become a global concern due to their persistent properties and impacts on the marine environment. This research investigates pollution sources and behaviors of microplastics at UNESCO Can Gio Mangrove Biosphere Reserve. Density flotation with sodium chloride is employed to extract microplastics from sand at Can Gio Beach, and a double‐filtration procedure is developed to recover microplastics from seawater at the beach and Dong Tranh Cape. The microplastics’ morphology and type are analyzed by micro‐Raman spectroscopy. The results show that microplastics are accumulated at concentrations from 31.99 to 92.56 MPs g^−1^ at various sand layers. The seawater at Can Gio Beach and Dong Tranh Cape contains 6.44 and 3.75 MPs L^−1^ of microplastics, respectively. White polyethylene fragments predominate, and all the microplastics comprise small secondary microplastics with a minimum size of 25 µm and a maximum size of 260 µm for fragments and a length of 640 µm for fibers. The proportions of polyethylene, polypropylene, polystyrene, and polymethylmethacrylate are similar. The differing percentages of other compositions in sand and seawater are attributed to the morphology and density of the microplastics. The results indicate the extent of microplastic pollution and suggest appropriate strategies for tourism development at the Biosphere Reserve.

## Graphical abstract

Microplastics in sand and seawater at Can Gio beach were determined by micro‐Raman spectroscopy. The result shows a correlation in composition of plastics in the two locations. Anthropogenic land‐based activities are the pollution source and microplastic distribution is affected by proximity to the source, tides, and the microplastics’ properties.

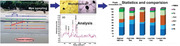

## 1 Introduction

Plastics are semi‐synthetic or synthetic polymers used to manufacture a wide range of products. Plastic goods are present in almost every aspect of our lives because of their low cost, light weight, durability, corrosion resistance and flexibility. The drastically increased worldwide production of plastics (over 300 million tons) has met a demand for disposable products, but the persistence of plastic waste has caused global ecological impacts.^[^ ^1^ ^]^ Plastic debris is ubiquitous in the marine environment and breaks into smaller pieces of various sizes, generally called microplastics (MPs). There is no official nomenclature for size classification, but MPs are restricted to pieces smaller than 5 mm with two subgroups: large MPs (1–5 mm) and small MPs (0.1–1 mm). Plastic debris originate mainly from land‐based activities, such as littering, tourism and insufficient waste management, but 10–25% of them come from sea‐based sources, particularly maritime transport, fishing and dumping.^[^ ^2^, ^3^ ^]^ It is estimated that 5 trillion plastic items, equivalent to 268 940 tons, are present in the world's oceans, of which MPs account for 92.4%.^[^ ^4^, ^5^ ^]^ The primary sources are purposely produced, such as bottles, containers, buoys, textile fibers, microbeads in cosmetics and even nanoparticles from industrial discharge. Depending on size, they are divided into macroplastics (2.5 cm to 1 m), mesoplastics (5 mm to 2.5 cm), micro and nanoplastics.^[^ ^5^ ^]^ Secondary MPs result from the breakdown and biochemical degradation of primary plastic debris under environmental weathering conditions.

MPs were first found in the North Atlantic in the 1970s.^[^ ^6^ ^]^ Due to their longevity, fragmentation and widespread distribution in the environment, the uptake and accumulation of MPs of various sizes in marine and terrestrial organisms have been reported.^[^ ^7^, ^8^, ^9^ ^]^ MPs, like other contaminants in the aquatic environment, can accumulate in sediments, so the MP concentration in sand can be an indicator of pollution in the adjacent ocean.^[^ ^10^ ^]^ The abundance and composition of plastic litter on beaches are determined not only by the distance from their sources but also by their fragmentation behaviors under beach hydrodynamic conditions.^[^ ^2^, ^11^, ^12^ ^]^ Despite the increasing number of studies since 2010 on MPs in diverse media, from environmental to biota samples, the sources of MPs as well as their behaviors in coastal ecosystems are not fully understood.^[^ ^13^ ^]^ Sand is a heterogeneous medium, and sand grains are redistributed under the effects of waves,^[^ ^14^ ^]^ tides and ocean currents, dispersing MPs in the sand layers and at various tidal lines.^[^ ^11^, ^15^, ^16^, ^17^ ^]^ The spatial distribution of MPs may be influenced by natural factors, such as wind, coastal landscapes and structures,^[^ ^18^ ^]^ as well as by anthropogenic factors, including river input, the local population and tourism.

Ho Chi Minh City (HCMC), also known as Saigon, is the largest and most populous economic centre of Vietnam. The aquatic environment has been seriously polluted by its increased population (9.98 million in 2014)^[^ ^19^ ^]^ and by the direct discharge of plastic waste into its urban canals every year. As a result, Saigon River carries at least 172 000 microfibers and 10 plastic fragments per cubic metre of water.^[^ ^20^ ^]^ The confluence of Saigon River and Dong Nai River flows to the East Sea through the Can Gio Mangrove Biosphere Reserve, the first biosphere reserve in Vietnam, designated by UNESCO in January 2000.^[^ ^21^ ^]^ Situated in an estuarine complex of tidal flats 50 km southeast of HCMC, it functions as a “green lung” of HCMC and a natural water filter for the marine ecosystem by absorbing carbon dioxide and receiving various pollutants in the rivers from HCMC on a daily basis.^[^ ^22^ ^]^ The rivers are driven by asymmetric semidiurnal tides in a tropical monsoonal climate with two distinct seasons: the dry season and the rainy season.^[^ ^23^ ^]^ The reserve is divided into three zones as regulated by UNESCO: 1) the core zone, with few households, is reserved for forest protection and scientific activities; 2) the buffer zone, where traditional exploitation of the rivers is unregulated; and 3) the transition zone, where tourism and intensive aquaculture (oysters, shrimps, clams, etc.) are being developed near coastal towns.^[^ ^21^, ^24^ ^]^ The transition zone ends with the free‐entrance Can Gio Beach. Tourism provides huge profits to the local people, but the daily use of plastic containers and tableware has contributed to environmental pollution in this zone.

The tide is one of the main external forces inducing changes to beach morphology.^[^ ^25^ ^]^ The seawater fluctuations generated by periodic tides move sediment, which disperses and redistributes MPs at the beach. Therefore, building a comprehensive beach sediment profile requires researching the distribution pattern of deposited materials at the sandy beach and studying the interactions of sand with other factors, such as human activities and the nearby seawater. Among reports on MPs in coastal areas,^[^ ^2^, ^5^, ^10^, ^11^ ^]^ there are few combined investigations of the distribution of plastic debris in sediment and seawater,^[^ ^26^, ^27^ ^]^ and the comparison and correlation pattern of plastic compositions in both media has not been well established. Research on MPs in coastal mangrove ecosystems remains sparse, and the studies have focused only on quantifying MPs in the sediment.^[^ ^28^, ^29^ ^]^ The authors’ literature search indicates that this study is the first to describe the occurrence of MPs in the Can Gio Mangrove Biosphere Reserve. The research aims to 1) investigate MP pollution in the sand at Can Gio Beach, 2) investigate MP pollution in seawater at the beach and a nearby location, and 3) evaluate the correlation between plastic compositions in both media at a specific tidal period. The information on the accumulation and distribution of MPs in the coastal ecosystem in this study will contribute to building a beach sediment profile of Can Gio Beach and to developing strategies to protect the biosphere reserve from plastic pollution.

## 2 Experimental Section

### 2.1 Sampling Procedure

**Figure 1:**
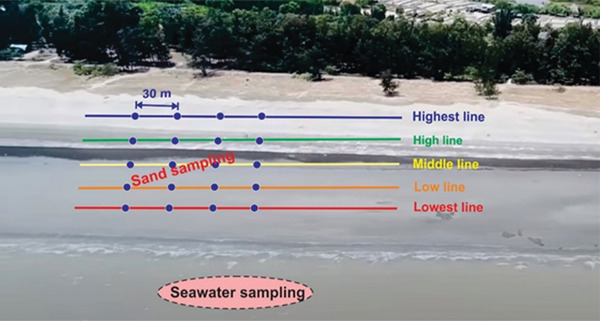
Sand and seawater sampling positions at Can Gio Beach.

The sampling was designed to investigate the effects of sand‐seawater interactions and human activities on the horizontal and vertical distribution of MPs at Can Gio Beach (10.3860 ^o^N, 106.9223 ^o^E). The sampling was conducted along five tidal lines in four sunny days with strong north‐easterly winds in the dry season of 2020. Each shoreline was divided into four equal distances on a transect of 120 m, and sand was collected at each position (**Figure** **1**). A square wooden quadrat measuring 50 × 50 and 2 cm high was pressed onto the sand layer to take surface samples (the top 2 cm). At low‐tide areas, the quadrat was positioned far enough from the waterline to prevent water intrusion into the quadrat, whilst dry sand in dunes was sometimes gently wetted with filtered seawater before digging. In addition, at the highest tidal line, the sand was dug to 5, 10, 15, and 20 cm with a stout shovel and held with piles of 5‐cm high quadrats. All materials within a 2‐cm quadrat were collected with a metal spoon, air‐dried and transported to the laboratory for analysis.

Seawater sampling was carried out at two locations: 1) at the location where sand was collected (10.3858 ^o^N, 106.9240 ^o^E) and 2) at Dong Tranh Cape (10.3777 ^o^N, 106.8782 ^o^E). The second location, 6 km from the beach, was an undeveloped area where local people from nearby households sometimes congregate and litter. The tourism beach clean‐up was carried out by food sellers, while there were no clean‐up operations at Dong Tranh. At each location, a total volume of 15 litres of bulk seawater was collected with a bucket for MP analysis.

### 2.2 Sample Preparation Procedure

Every liter of seawater was firstly filtered through a 47‐mm glass‐fiber membrane with 1.6‐µm pore size (GF/A, Whatman, GE Healthcare) with the aid of vacuum pump. Visible items on the filter paper were picked up, cleaned with filtered deionized water (FDW) and stored for direct analysis. The remaining materials containing potential small microplastics were washed off with FDW to the final volume of about 100–200 mL. The supernatant was then oxidized with 30% hydrogen peroxide at 70–80 °C until the solution was discolored and the mixture was incubated at room temperature for 24 h. Finally, MPs were separated by filtering the solution and analyzed by Raman microscope.

All sand samples were dried at 50 °C until the weight remained unchanged. Non‐plastic items such as shells, moss, pebbles in sand samples were picked up and washed carefully with FDW onto a GF/A paper to collect micro‐pieces attached to them. Non‐plastic items were discarded and micro‐pieces were kept for analysis. A density flotation method as described in Hidalgo‐Ruz et al. (2021) was employed to extract microplastics from sand.^[^ ^30^ ^]^ A weight of 100 g sand was soaked in 300 mL of filtered saturated sodium chloride solution (1.264 g cm^−3^), stirred for 30 min and let static for some hours at the room temperature (30 °C ± 5). The floating items were picked using metal forceps, and stored in Petri dishes for plastic analysis. The supernatant above the sand layer was transferred into a second glass beaker. A volume of 10 mL 30% H_2_O_2_ was added and stirred at 80°C ± 5 until the solution was discolored, then incubated overnight in dark at room temperature. The solution was then filtered through a GF/A membrane. The filters were air dried for 24 h and sealed in Petri dishes for analysis of invisible microplastics. There were three replications for each sampling position.

### 2.3 Microplastic Analysis

The MPs analysis was subject to determine individual particles by using a Raman microscope (XploRA Horiba Scientific). The microscopic mode was used to observe shape, color and size of micro‐pieces and the number of microplastics with the total dimensions larger than 25 µm was noted down. The Raman spectrometer included a single beam laser operating at 532 nm of wavelength coupled with a charge‐coupled device detector. Every individual particle was analyzed in a wavelength range of 50–3600 cm^−1^ with an acquisition time of 15 s and a grating of 900 lines per mm. After identifying the polymer type based on Raman spectra, microplastics were categorized according to shape, polymer type and color.

The deionized water and saline solutions were filtered before used for washing steps. The air control samples were clean filter membranes on glass Petri dishes around the working area during the sampling handling and vacuum filtering stage. No microplastics were found on the controls, suggesting that fibers encountered in the study were not from clothing used in the laboratory.

### 2.4 Statistical Analysis

One‐way analysis of variance (ANOVA) was used to establish differences in MPs abundance between sand positions at each tidal line. A Turkey's multiple comparison test was used to analyze the mean differences between the total microplastic amounts in each sand position sampling. All statistical analyses were performed at 95% confidence interval on SPSS v22 software.

## 3 Results and Discussion

### 3.1 Microplastic Pollution in the Sand at Can Gio Beach

#### 3.1.1 Spatial Variation in the Microplastic Amounts along the Tidal Lines

**Figure 2:**
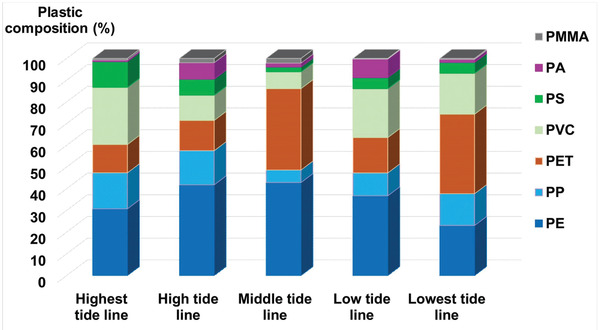
The distribution of plastic components at different tidal lines.

A total of 523 MP pieces were analyzed for polymer type using Raman spectroscopy. As this technique cannot distinguish between high‐density and low‐density polyethylene (HDPE and LDPE, respectively),^[^ ^5^ ^]^ such pieces were categorised only as polyethylene (PE). PE accounted for the highest proportion, from 30.72% to 42.84%, in almost all the sand samples (**Figure** **2**). It is also the most abundant component at Vung Tau and Tien Giang beaches in Vietnam,^[^ ^31^ ^]^ in Taiwan,^[^ ^10^, ^32^ ^]^ Hong Kong,^[^ ^33^ ^]^ the Bohai Sea (China),^[^ ^34^ ^]^ in the Northern Gulf of Mexico Estuary^[^ ^4^ ^]^ and at Kamilo and Kahuku beaches in Hawai'i.^[^ ^5^ ^]^ Although polypropylene products are popular as disposable bottle caps, cups and straws, this polymer is not predominant in this area compared to other places.^[^ ^4^, ^33^ ^]^ Polyethylene terephthalate (PET) was the second largest group (23.47%) and appeared as coloured fibers and white and pink fragments. Other polymers from single‐use products include Poly(vinyl chloride) (PVC), polypropylene (PP), and polystyrene (PS), and they were all encountered in this study area, with average percentages of 12.62%, 15.11%, and 6.3%, respectively. Poly(methyl methacrylate) (PMMA) is not a common material in plastic household products, so it was always found in the smallest quantities. In some cases, Raman signals cannot precisely recognise the chemical components of dark‐coloured items because of melting or burning phenomena when the laser beam's energy is absorbed by the sample.^[^ ^5^ ^]^ Therefore, 31 items of homogenous shape and clear colour were regarded as unidentified MPs. They included fibers in dark colours, such as brown, black, grey and blue, and fragments with fouling or pitting.

The amount of MPs in the sand decreased or increased from the dune towards the sea (**Table** **1**). It has been reported that MPs are found in the largest quantity on the beach at the high tide line,^[^ ^11^, ^17^ ^]^ but that finding must be qualified, as ANOVA shows no statistically significant differences between different tidal lines of each position (Table S2, Supporting Information), despite fewer MPs being found at the water‐edge line. One hypothesis is that MPs are transported and broken under the force of waves into small fragments, allowing them to drift and redeposit offshore.^[^ ^33^ ^]^ A second possibility is that plastic debris experience photooxidative breakdown in the dune under favourable conditions of temperature and sunlight, after which their fragmented products are transported to the sea. Indeed, the microfibers at the water‐edge lines (38.93–183.47 µm) were much shorter than those at the upper shorelines (80–438.25 µm). The second hypothesis is partially true, as many small MPs can be buried in deep layers of sand by human activity on the beach prior to being transported.^[^ ^11^, ^34^ ^]^

**Table 1:** The two‐dimensional spatial distributions of microplastics on sandy beach

Sampling position^a)^	Position 1	Position 2	Position 3	Position 4	Average in line
Highest tide line (1)	74.96 ± 19.17	38.95 ± 4.37	68.32 ± 38.08	67.61 ± 15.53	62.46 ± 10.58
High tide line (2)	72.80 ± 16.37	43.81 ± 12.70	84.73 ± 21.00	65.64 ± 15.17	67.10 ± 7.25
Middle tide line (3)	59.92 ± 21.53	49.41± 5.35	66.97 ± 18.74	84.26 ± 5.40	61.05 ± 8.90
Low tide line (4)	86.37 ± 5.14	58.03 ± 15.58	59.26 ± 5.09	31.99 ± 6.64	58.91 ± 6.45
Lowest tide line (5)	77.02 ± 18.44	53.70 ± 12.33	58.09 ± 4.29	80.14 ± 20.38	70.33 ± 8.88

^a)^ The value of microplastics abundance is (mean ± std. error) MPs kg^−1^.

The ANOVA analysis of MP amounts did not find statistically significant differences between the sand positions at each tidal line except at the low tide line (*p* = .01) (Table S3, Supporting Information). A vast amount of MP was found in the tourist gathering areas: 74.96 MPs kg^−1^ in position 1 of line 1 and 72.98 MPs kg^−1^ in position 1 of line 2. Surprisingly, however, the amount of MP was very low in position 2 of line 1 (38.95 MPs kg^−1^) and line 2 (43.81 MPs kg^−1^), even though there were several tourists there. The difference was caused mainly by various human activities in the dune rather than by the waves, as the sand was very dry and the seawater did not reach these positions during daylight hours. A Tukey's comparison test found the specific difference in MP amount between positions 1 and 4 in line 4 at *p* = .006 (Table S4, Supporting Information). Indeed, the MP fragments were concentrated mainly in position 1, and their number decreased in positions 2 and 3. At position 4, the farthest position from the tourist gathering area, the number of MPs was lowest at only 31.98 MPs kg^−1^.

#### 3.1.2 The Abundance and Composition of Microplastics Accumulated in Various Sand Layers

**Figure 3:**
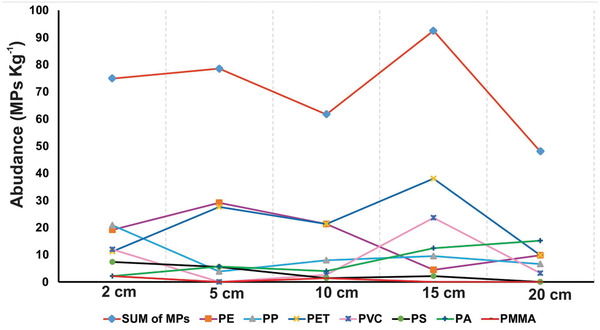
Microplastic accumulation in different sand layers.

**Figure** **3** shows the changes in amount of total microplastics and each plastic type in different depths. It was observed that surface samples (the top 0–5 cm) contain higher numbers of MPs than deeper layers, similarly to previous research.^[^ ^16^, ^31^, ^35^ ^]^ There is a slight decreasing trend in the abundance of total MPs and some plastic types with increasing depth of sand. The surface sand layer exchanges components directly with the seawater, resulting in more MPs being retained in the top layers than in samples beneath the surface. Additionally, the surface directly receives plastic waste, which is broken into smaller fragments by tourist activities. However, the ANOVA found no statistically significant difference in the total MPs in the sand layers (*p* = 0.05) (Table S5, Supporting Information), as was also observed in the Bohai Sea.^[^ ^34^ ^]^ Moreover, a vast quantity of small plastic fragments was encountered at a depth of 15 cm (up to 92.45 MPs kg^−1^). This shows that small MPs were not easily removed by seawater or beach clean‐up when they were buried in the sand sediment.^[^ ^11^ ^]^

**Figure 4:**
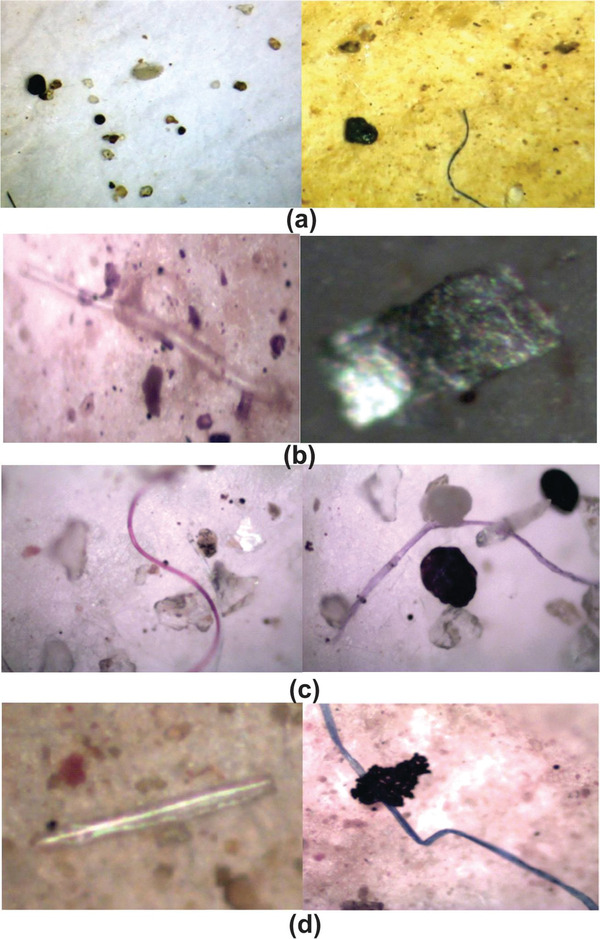
Microplastics commonly found in sand at different depths: a) Low tidal lines; b) 2‐cm and 5‐cm top: PE translucent bar (left picture) and fragment (right picture); c) 15 cm: PA pink, purple fibers, PET white fragment (left picture); d) 20 cm: PVC white stick (left picture), PA blue fiber (right picture).

Although the MPs in the various layers did not vary remarkably, the distribution trend of each plastic type differed. PE was the dominant type from the top layers to 10 cm deep (26–36%), while PET predominated in the lower layers. PS boxes were seen on the beach, but they were not counted as MPs due to their large size (>5 mm). Nylon‐6 appeared in deep sand layers as coloured microfibers (purple, blue and pink) ranging in length from 115 to 250 µm. They were entangled with other plastic fragments and sand grains in 15‐cm deep samples (**Figure** **4**c). The entanglement may provide a feasible explanation of why this polymer was retained in deep sand layers. In contrast to long fibers, PET, PVC, and polyamide (PA) fragments tended to accumulate in the deep layers, possibly due to their tiny sizes (25–70 µm) and high density.^[^ ^33^, ^36^ ^]^

The shape and colour of MPs to some extent show their degradation. The fragmentation is caused by mechanical forces, such as sand abrasion or wave action. More importantly, photochemical weathering occurs when MPs are exposed to heat and ultraviolet (UV) light. The climate at Can Gio is tropical monsoon, and the monthly average temperature ranges from 25.8 to 29 °C.^[^ ^22^ ^]^ The high temperatures and 5–9 sunshine hours every day create ideal conditions for plastic fragmentation,^[^ ^23^ ^]^ especially in the upper sand layers. Long‐term exposure to high‐intensity sunlight makes plastic particles weak, brittle and susceptible to damage.^[^ ^36^ ^]^ As a result, almost all the pieces were fragments in the size range of 30–135 µm, with rounded corners or jagged corners. Additionally, their colours were affected. Many fragments were found to be translucent (Figure 4b), which could be caused by the discolouring action of UV light.^[^ ^32^, ^36^ ^]^ Other fragments were milky white, blue, red or orange.

### 3.2 Microplastic Pollution in Seawater at Can Gio Beach and Dong Tranh Cape

The abundance of microplastics in the seawater at Can Gio Beach (6.44 ± 2.98 MPs L^−1^) was higher than in the nearby town (Dong Tranh Cape, 3.75 ± 2.04 MPs L^−1^). PE was the most abundant polymer, and it was found mainly in non‐fibrous forms, especially fragments (Figure S1, Supporting Information). There was not big difference in PP amount in Dong Tranh and beach seawater which was 0.47 and 0.75 MPs L^−1^, respectively. PS with relatively high density (1.04–1.1 g cm^−3^) can be made into hard products such as packaging, toys, car parts.^[^ ^37^, ^38^ ^]^ Also, this material can be combined with air to make foam‐like products used for surfboards, cups, and boxes. Styrofoam bubbles were hardly caught due to their buoyancy, floating and stranding abilities under the blowing force of winds and waves.^[^ ^11^ ^]^ Since the sampling occurred under condition of strong winds, a very small number of white PS foams were identified compared to other polymer types in beach seawater. Similarly, there was no PS composition in Dong Tranh seawater.

**Figure 5:**
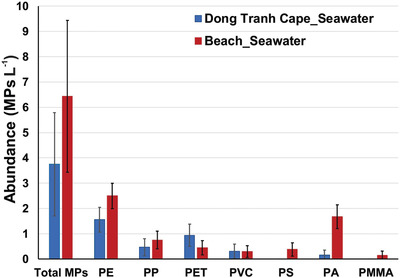
Microplastic abundance in seawater at two study areas.

The incidence of other plastics was noticeably different in these two areas (**Figure** **5**), indicating that the contamination came not only from beach seawater but also from other water areas, as Dong Tranh Cape is barren sand at the sea estuary. Only two of 10 separate fibers were identified as nylon in the Dong Tranh seawater; by contrast, 22 nylon items were filtered from the beach seawater, nine of them appearing as coloured fibers (Figure S1a, Supporting Information). This difference reveals the effects of human activity on the nearby sand. A higher number of PA fibers may be released directly from the swimwear of tourists into the beach seawater,^[^ ^10^ ^]^ compared to a very small number of microfibers transported from the washing activities of the local people near Dong Tranh Cape. Conversely, only a small number of white PET fragments were observed in the beach seawater, while it was the second most abundant polymer in the Dong Tranh seawater.

### 3.3 Comparison of Microplastic Pollution between Seawater and Beach Sand

**Figure 6:**
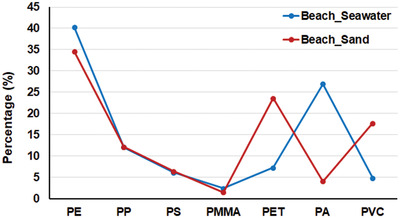
Microplastic compositions in seawater and sand at Can Gio Beach.

The discrete investigations of MPs in sand and seawater showed the similarity in MP compositions in the two environments. PE was the predominant component in both seawater and sand (**Figure** **6**), and most of this polymer appeared as small white fragments. The percentage of PE in seawater was slightly higher than in sand, and, notably, the average size of PE pieces in seawater was smaller (**Table** **2**). This indicates that tiny PE particles with low density are washed out more easily from the sand than larger particles under tidal action. The same trend was found in the PP, PS and PMMA proportions in sand and seawater.

**Table 2:** Comparisons of microplastics extracted from sand and seawater

Criteria	MPs in beach sand	MPs in beach seawater
Total MPs	60.22 ± 19.85 MPs kg^−1^	6.44 ± 2.98 MPs L^−1^
MPs compositions	PE (34.61%) PP (12.13%) PS (6.42%) PET (23.59%) PA (4.04%)	PE (40.32%) PP (12.10%) PS (6.13%) PA (26.93%) PET (7.26%)
Size range (µm)	Fragment: 32.5–260 Fiber: 25.12–640.11	Fragment: 25–101 Fiber: 26–599.93
Color	White (55%), black (23.64%), pink (10.91%), purple (6.36%), blue (4.09%)	White (48.04%), back (32.35%), blue (12.74%), pink (4.9%), purple (1.96%)

PET was the second most abundant MP in beach sand. Many single‐use plastic products are made of this polymer, for instance, water bottles, food and beverage packaging,^[^ ^37^, ^39^ ^]^ dispensing containers and biscuit trays.^[^ ^40^ ^]^ Furthermore, the world's most common PET production is for synthetic fibers, also known as polyester.^[^ ^39^ ^]^ Thus, the predominantly PET and PA coloured fibers possibly came from textile sources, such as swimsuits or UV‐light resistant clothing, while non‐fibrous MPs of PET and other polymers (PE, PP, PVC and PS) indicate a food‐packaging origin.^[^ ^39^, ^40^ ^]^ Nevertheless, the amount of PET in beach seawater indicates that PET pieces do not easily detach from the sand layers. The deposition of MPs is also influenced by the dissipation of a wave when it reaches the shore.^[^ ^33^, ^41^ ^]^ Hence, some fragments were transported to the sea, while others remained. All the PET fragments filtered from seawater were quite small (27–45 µm) compared to those found in the sand (28–197.5 µm), and the fibers were longer (Figure S1a, Supporting Information) (144.81 µm on average) than those extracted from the sand (Figure S1c, Supporting Information) (long fibers of over 105 µm). It is reasonable to believe that tiny particles would be blown easily and therefore dispersed widely in seawater.

The findings for PVC accumulation were similar to those for PET fragments. PVC is also a dense polymer, so PVC debris tended to be retained in sand layers rather than transported to the seawater.^[^ ^32^ ^]^ The amount of PVC was high in the sand samples but very low in seawater, which shows that this polymer tends to accumulate in the sand sediment^[^ ^32^ ^]^ but sink to the bottom in seawater, as its density (1.15–1.70 g cm^−3^) is higher than that of seawater (1.023 g cm^−3^).^[^ ^36^ ^]^ Hence, only light fragments floating at the surface layer could be filtered and identified. Because industrial manufacturing is not allowed within the reserve, the PVC is assumed to be fragmentation products of clothing, shopping bags and food packaging.^[^ ^38^ ^]^

A higher number of polyamides was identified in beach seawater than in beach sand. Blue, purple, pink, white and black were common colours of the fibers in beach seawater, but pink fibers were not seen in the Dong Tranh seawater. PA accounted for a very low percentage in the sand at high tidal lines (lines 1, 2, and 3), but the amount was higher in sand at the low tidal line (line 4) (Figure 2). Few PA fibers were found at the lowest line (line 5). Beach seawater was taken at line 5, and the huge amount of coloured fibers in beach seawater was dominated by PA (Figure 6). This clearly suggests that pollution may be caused directly by swimming, as the MP fibers could be shed from the swimsuits and clothes of tourists.^[^ ^2^ ^]^ The fibers ranged from 35 to 459.19 µm; they may enter the water environment directly and then follow the oceanic currents to return to the sand.

The results reveal the sources of MP pollution at this shoreline, which depend on how the MPs enter the water.^[^ ^42^ ^]^ Contamination from the seawater to the beach sand was unlikely because of the geographical location of the study area. Firstly, the rivers traverse a mangrove forest of 75 740 hectares before reaching this zone. The abundance of MPs was much lower in the seawater at Can Gio Beach (7 MPs L^−1^) than in the Saigon–Dong Nai (SG‐DN) river estuary system (22–251 MPs L^−1^).^[^ ^43^ ^]^ This significant difference reflects the dispersion of MPs over a distance of 50 km and the role of mangroves in keeping contaminants from the marine environment. Secondly, this location is not a waterway junction of marine traffic in the South China Sea, and there is no ocean dumping here. Thus, the sea‐based sources of plastic waste could be negligible. Furthermore, the total number of MP pieces in the adjacent water regions, for example, Dong Tranh Cape, was not high enough to contaminate the sandy beach.

Compared with other beaches, with the exception of the southern Baltic Sea (0–53 MPs kg^‐1^),^[^ ^13^ ^]^ the total of MPs (60.22 ± 19.85 MPs kg^−1^) at this sandy beach is not high. In Vietnam, the total is actually lower than at the Vung Tau tourist beach (0–295 MPs kg^−1^)^[^ ^31^ ^]^ due to UNESCO's preservation regulations and the low population density of the whole Can Gio district (only 110 inhabitants km^−2^). The MPs in SG‐DN were mainly fibers,^[^ ^20^ ^]^ while fragments dominated in the Can Gio Beach ecosystem, and almost all of them were PE, a thermoplastic material for single‐use products, such as utensils, water and milk bottles, bags, ice cream and fast food containers.^[^ ^37^, ^40^ ^]^ These facts show that the pollution sources presumably the recreation activities of the tourists at the beach must be closely associated with this location, as non‐mangrove related fishing activities are not popular at Can Gio Beach.^[^ ^22^ ^]^ This conclusion supports the belief that MP distribution is impacted by population density, proximity to urban areas^[^ ^26^ ^]^ and anthropogenic activities.^[^ ^40^, ^41^ ^]^

The presence of MPs in a beach ecosystem is mainly governed by water circulation^[^ ^27^ ^]^ and is affected by the morphology and density of the MPs. The research found similar percentages of colours, shapes and size ranges of MPs in seawater and sand (Table 2), which aligns with the data of Zhu et al. (2018) in the northern Yellow Sea (China).^[^ ^41^ ^]^ Regarding the correlation patterns of the distribution of plastic types in seawater and sand environments, the percentage of PE in seawater is higher than in beach sediment as also described in Zhu et al. and Sagawa et al.^[^ ^41^, ^44^ ^]^ Nevertheless, nylon makes up a higher percentage in seawater than in beach sand, which is the reverse of the finding of Zhu et al. (2018).^[^ ^41^ ^]^ Therefore, further investigations are merited in the future to reach a comprehensive conclusion.

Single sampling was implemented by taking samples on four consecutive days at the same time every day so that the results would not be influenced by the weather and tidal pattern; samples taken at various times would not provide consistent data between days due to differences in the tidal pattern, resulting in uncertain conclusions about the correlation of plastic composition between the seawater and sand environments. However, this sampling strategy has a limitation. The result represents one month in the dry season of the year, and the finding would change if the samples were collected at different times, quarters or years because the tidal time varies from the dry season to the rainy season. We consider this investigation a pilot study because, to our best knowledge, 1) no similar study has been conducted before in Can Gio and 2) the technical experience gained in this investigation will enable us to work more efficiently when the sample size is increased to investigate the temporal MP distributions and relationship patterns of plastic composition in the beach ecosystem in the future.

## 4 Conclusions

This is the first study on MPs in seawater and the sandy beach at the Can Gio Mangrove Biosphere Reserve. The total MP abundance provided a general overview of plastic pollution in this region. The mean concentrations were 60.22 ± 19.05 MPs kg^−1^ in all the sampled sand and 6.44 ± 2.98 and 3.75 ± 2.04 MPs L^−1^ in the seawater at the sandy beach and Dong Tranh Cape, respectively. The variety in plastic composition revealed the anthropogenic sources of pollution in the transition zone of the reserve. The occurrence of MPs in the seawater results not only from human activities ashore but also from water movement in the marine environment. The water circulation bring contaminants from adjacent areas and disperses some of them in the ocean. The comparison of MPs in the seawater and the sandy beach indicated that land‐based activities were the main pollution sources at the coastal environment. PE was the dominant polymer because of single‐use plastic products associated with reactional activities on the beach. PET and polyamide were also common polymers and were identified in diverse morphologies, especially coloured fibers and white particles, as a result of direct shedding from tourists’ clothes and the breakdown of food packaging waste. The most abundant MP shape (40–88%) was fragments, commonly in small sizes (25–100 µm), suggesting a secondary MP source from the degradation of plastic waste on the beach. The similarities and differences in plastic distribution reveal their migrations between two kinds of environment when superficial sand layers exchange their materials with seawater. Light polymers, such as PE and PP particles, were easily carried by winds and tidal currents at the beach, whereas denser polymers, such as PET and PVC, tended to accumulate in the sand layers. The UNESCO Biosphere Reserves have been set aside not only for habitat conservation and biological diversity but also to support research and the economic development of the local community. Local people have benefited from the mangrove ecosystem for decades and have polluted the marine environment to an extent. Recently, the exponential growth of tourists in a limited‐capacity beach has increased noise and light pollution as well as solid and liquid waste, resulting in the presence of plastic debris in sand sediments and seawater. This study provides a useful data source for managing pollution and developing appropriate strategies for tourism development combined with mangrove biosphere reserve protection.

## Conflict of Interest

The authors declare no conflict of interest.

## Supporting information

Supporting Information

## Data Availability

The data that supports the findings of this study are available in the supplementary material of this article.
