# Supplementary material for: Comparison of Microplastic Pollution in Beach Sediment and Seawater at UNESCO Can Gio Mangrove Biosphere Reserve
Source: Glob Chall. 2021 Jul 16;5(11):2100044. doi: 10.1002/gch2.202100044 (PMC8562063; doi:10.1002/gch2.202100044)
Supplement: Supplementary file 1 — Supporting Information [file GCH2-5-2100044-s001.pdf]

© 2021 Wiley-VCH GmbH

Global Challenges Open Access

Supporting Information

for *Global Challenges,* DOI: 10.1002/gch2.202100044

Comparison of Microplastic Pollution in Beach Sediment and Seawater at UNESCO Can Gio Mangrove Biosphere Reserve

Vo Thi Kim Khuyen, Dinh Vu Le,* Axel René Fischer, and Christina Dornack*

((Supporting Information can be included here using this template))

Supporting Information

Comparisons of microplastic pollution between seawater and sandy beaches in Can Gio Mangrove Biosphere Reserve

Vo Thi Kim Khuyen[1], Dinh Vu Le[2*], Axel René Fischer[1], Christina Dornack[1]*

Table S1. Microplastic distribution in sand from highest line to lowest line

Highest tide line (LINE 1) (onshore)	Sample S4 (Position 4)	Sample S3 (Position 3)	Sample S2 (Position 2)	Sample S1 (2 cm) (Position 1)
	40	28.12	32.59	67.96
	93.72	32.41	36.94	45.81
	69.12	144.44	47.32	111.11
High tide line (LINE 2) (on the beach)	Sample 4.1 (Position 4)	Sample 3.1 (Position 3)	Sample 2.1 (Position 2)	Sample 1.1 (Position 1)
	100	117.95	68.43	112.12
	77.82	45.87	26.08	45.81
	55.56	90.38	36.92	87.45
	29.18			45.81
Middle tide line (LINE 3) (on the beach)	Sample 4.2 (Position 4)	Sample 3.2 (Position 3)	Sample 2.2 (Position 2)	Sample 1.2 (Position 1)
	74.91	40	40	133
	93.63	103	58.52	40
	84.25	57.91	49.72	20
				46.68
				13.01
Low tide line (LINE 4) (on the beach)	Sample 4.3 (Position 4)	Sample 3.3 (Position 3)	Sample 2.3 (Position 2)	Sample 1.3 (Position 1)
	39.27	65.02	38	80
	13.09	58.62	85.2	97.72
	32.72	45.2	84	92.1
	42.87	68.21	24.9	75.65
Lowest tide line (LINE 5) (towards the sea)	Sample 4.4 (Position 4)	Sample 3.4 (Position 3)	Sample 2.4 (Position 2)	Sample 1.4 (Position 1)
	60	51.91	89.43	140
	47.1	69.23	32.83	105.65
	164.08	50.78	45.29	120
	125.4	60.42	47.23	64.93
	67.7			38
	96.71			70.56
	0			0

Table S2. ANOVA analysis for 5 lines at each position

Position 1:

Summary

Groups	*Count*	*Sum*	*Average*	*Variance*
Column 1	3	224.88	74.96	1102.773
Column 2	4	291.19	72.7975	1072.535
Column 3	5	252.69	50.538	2316.709
Column 4	4	345.47	86.3675	105.7169
Column 5	7	539.14	77.02	2379.33

ANOVA

Source of Variation	*SS*	*df*	*MS*	*F*	*P-value*	*F crit*
Between Groups	3333.718	4	833.4296	0.5123	0.727533	2.927744
Within Groups	29283.11	18	1626.84			
Total	32616.83	22				

Position 2:

Summary

Groups	*Count*	*Sum*	*Average*	*Variance*
Column 1	3	116.85	38.95	57.2733
Column 2	3	131.43	43.81	483.9847
Column 3	3	148.24	49.41333	85.81813
Column 4	4	232.1	58.025	970.4825
Column 5	4	214.78	53.695	608.2596

ANOVA

Source of Variation	*SS*	*df*	*MS*	*F*	*P-value*	*F crit*
Between Groups	791.9406	4	197.9851	0.396606	0.80734	3.259167
Within Groups	5990.378	12	499.1982			
Total	6782.319	16				

Position 3:

Summary

Groups	*Count*	*Sum*	*Average*	*Variance*
Column 1	4	204.97	51.2425	4066.96
Column 2	3	254.2	84.73333	1322.795
Column 3	3	200.91	66.97	1053.813
Column 4	4	237.05	59.2625	103.7911
Column 5	4	232.34	58.085	73.71897

ANOVA

Source of Variation	*SS*	*df*	*MS*	*F*	*P-value*	*F crit*
Between Groups	2168.616	4	542.1539	0.403051	0.80314	3.179117
Within Groups	17486.63	13	1345.125			
Total	19655.24	17				

Position 4:

Summary

Groups	*Count*	*Sum*	*Average*	*Variance*
Column 1	3	202.84	67.61333	723.1621
Column 2	4	262.56	65.64	919.9667
Column 3	3	252.79	84.26333	87.60973
Column 4	4	127.95	31.9875	176.3719
Column 5	7	560.99	80.14143	2907.232

ANOVA

Source of Variation	*SS*	*df*	*MS*	*F*	*P-value*	*F crit*
Between Groups	7014.975	4	1753.744	1.255255	0.327907	3.006917
Within Groups	22353.95	16	1397.122			
Total	29368.92	20				

Table S3. ANOVA analysis for 4 positions at each tide line

Highest tide line (LINE 1):

Summary

Groups	Count	Sum	Average	Variance
Column 1	3	224.88	74.96	1102.773
Column 2	3	116.85	38.95	57.2733
Column 3	4	204.97	68.32	4066.96
Column 4	3	202.84	67.61333	723.1621

ANOVA

Source of Variation	SS	df	MS	F	P-value	F crit
Between Groups	2410.006	3	803.3353	0.452802	0.721655	3.862548
Within Groups	15967.3	9	1774.144			
Total	18377.3	12				

High tide line (LINE 2):

Summary

Groups	Count	Sum	Average	Variance
Column 1	4	291.19	72.7975	1072.535
Column 2	3	131.43	43.81	483.9847
Column 3	3	254.2	84.73333	1322.795
Column 4	4	262.56	65.64	919.9667

ANOVA

Source of Variation	SS	df	MS	F	P-value	F crit
Between Groups	2698.448	3	899.4827	0.937834	0.458254	3.708265
Within Groups	9591.065	10	959.1065			

Total	12289.51	13

Middle tide line (LINE 3):

Summary

Groups	Count	Sum	Average	Variance
Column 1	5	252.69	59.928	2316.709
Column 2	3	148.24	49.41333	85.81813
Column 3	3	200.91	66.97	1053.813
Column 4	3	252.79	84.26333	87.60973

ANOVA

Source of Variation	SS	df	MS	F	P-value	F crit
Between Groups	2680.462	3	893.4874	0.762276	0.540556	3.708265
Within Groups	11721.32	10	1172.132			
Total	14401.78	13				

Low tide line (LINE 4):

Summary

Groups	Count	Sum	Average	Variance
Column 1	4	345.57	86.3675	105.7169
Column 2	4	232.1	58.025	970.4825
Column 3	4	237.05	59.2625	103.7911
Column 4	4	127.95	31.9875	176.3719

ANOVA

Source of Variation	SS	df	MS	F	P-value	F crit
Between Groups	5918.571	3	1972.857	5.818083	**0.010811**	3.490295
Within Groups	4069.087	12	339.0906			
Total	9987.658	15				

Lowest tide line (LINE 5):

Summary

Groups	Count	Sum	Average	Variance
Column 1	7	539.14	77.02	2379.33
Column 2	4	214.78	53.695	608.2596
Column 3	4	232.34	58.085	73.71897
Column 4	7	560.99	80.14143	2907.232

ANOVA

Source of Variation	SS	df	MS	F	P-value	F crit
Between Groups	2693.795	3	897.9315	0.47868	0.70111	3.159908
Within Groups	33765.3	18	1875.85			
Total	36459.1	21				

Table S4. Turkey's comparison test result for Line 4

ANOVA

Mps

	Sum of Squares	df	Mean Square	F	Sig.
Between Groups	5918.571	3	1972.857	5.818	.011
Within Groups	4069.087	12	339.091		
Total	9987.658	15			

Multiple Comparisons

Dependent Variable: Mps

Tukey HSD

(I) P	(J) P	Mean Difference (I-J)	Std. Error	Sig.	95% Confidence Interval	
					Lower Bound	Upper Bound
Position 1	Position 2	-27.27500	13.02096	.210	-65.9329	11.3829
	Position 3	-26.03750	13.02096	.241	-64.6954	12.6204
	Positon 4	-54.38000*	13.02096	.006	-93.0379	-15.7221
Position 2	Position 1	27.27500	13.02096	.210	-11.3829	65.9329
	Position 3	1.23750	13.02096	1.000	-37.4204	39.8954
	Positon 4	-27.10500	13.02096	.214	-65.7629	11.5529
Position 3	Position 1	26.03750	13.02096	.241	-12.6204	64.6954
	Position 2	-1.23750	13.02096	1.000	-39.8954	37.4204
	Positon 4	-28.34250	13.02096	.185	-67.0004	10.3154
Positon 4	Position 1	54.38000*	13.02096	.006	15.7221	93.0379
	Position 2	27.10500	13.02096	.214	-11.5529	65.7629
	Position 3	28.34250	13.02096	.185	-10.3154	67.0004

*. The mean difference is significant at the 0.05 level.

Table S5. ANOVA analysis for 5 depth samples

Summary

Groups	Count	Sum	Average	Variance
Column 1	3	224.88	74.96	1102.773
Column 2	4	314.35	78.5875	145.2013
Column 3	5	308.59	61.718	1844.831
Column 4	4	370.27	92.5675	160.804
Column 5	4	192.71	48.1775	209.4719

ANOVA

Source of Variation	SS	df	MS	F	P-value	F crit
Between Groups	4647.965	4	1161.991	1.565843	0.234337	3.055568
Within Groups	11131.3	15	742.0867			

Total	15779.27	19

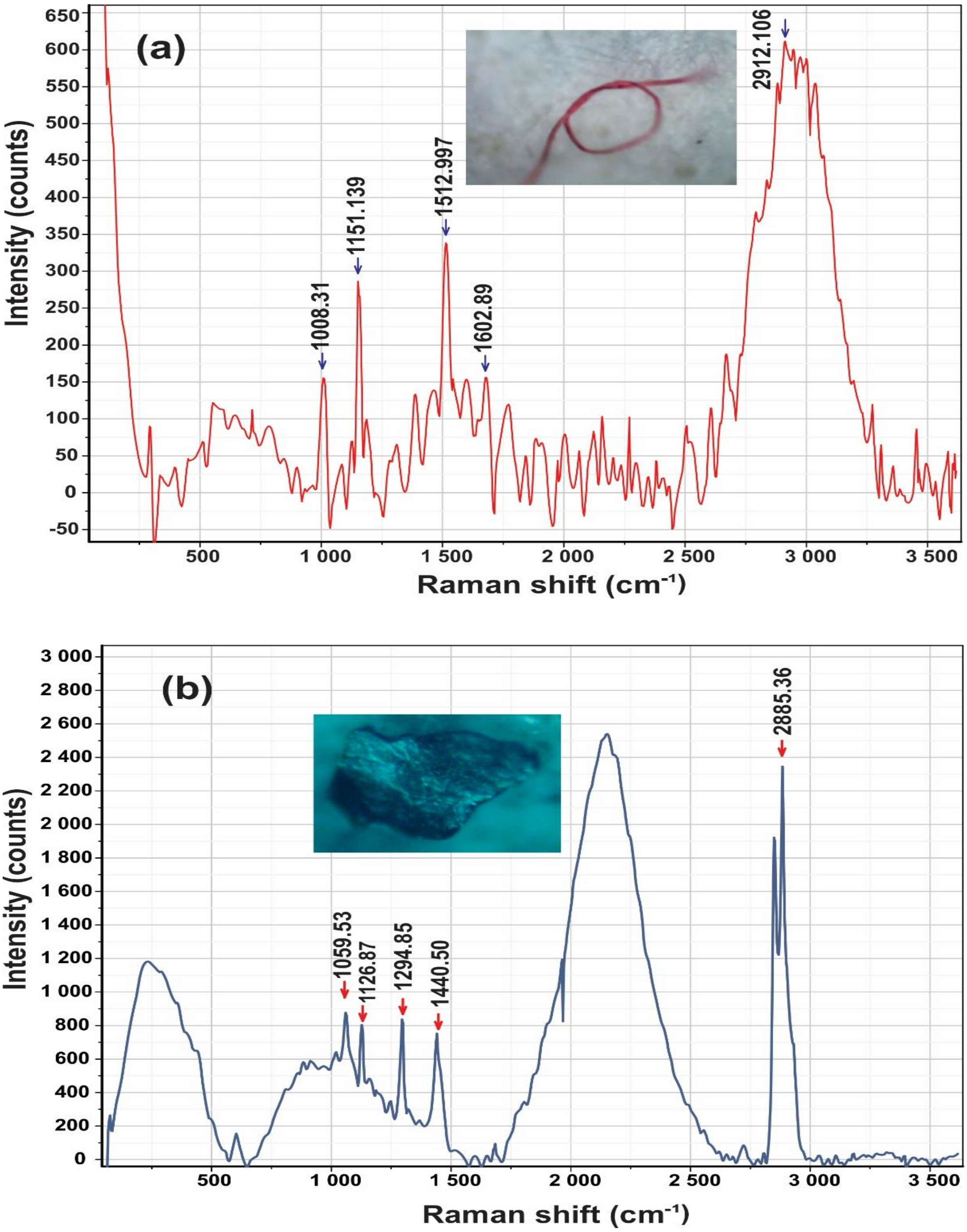

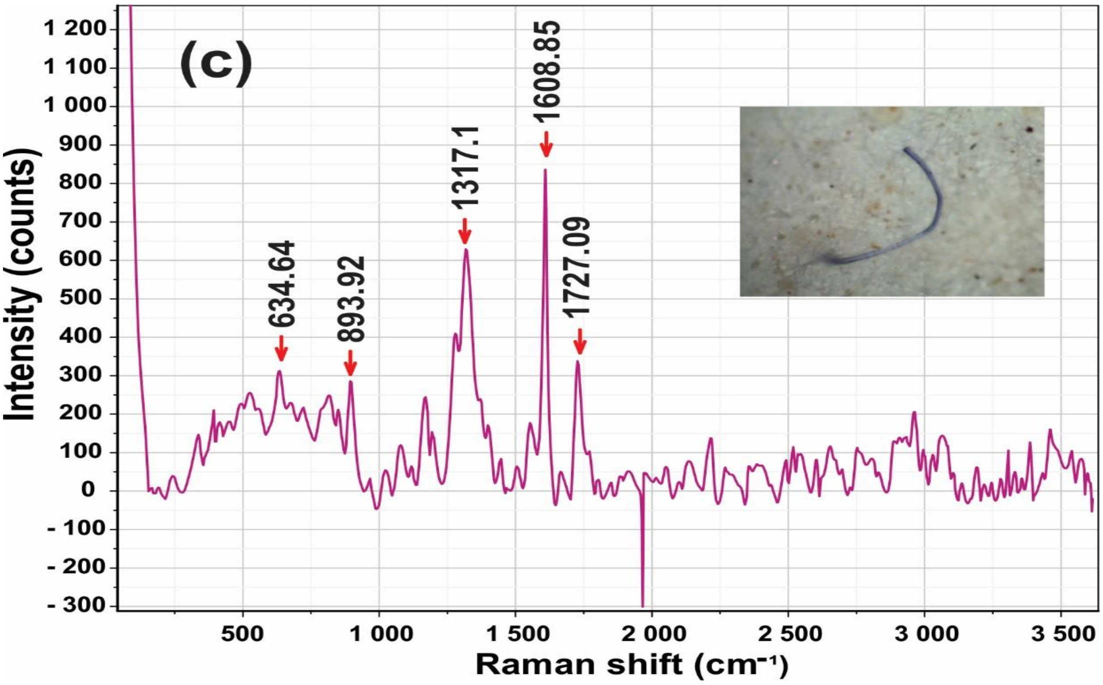

Figure S1. Typical microplastics commonly found in the study area. PA long pink fiber in beach seawater (a), PE green fragment in Dong Tranh seawater (b), PET short blue fiber in sandy beach (c).
